# Direct Integration of Electrospun Polycaprolactone Fibers with Polydimethylsiloxane: Influence of Elastomer Formulation on Fiber–Elastomer Adhesion

**DOI:** 10.3390/ijms27198785

**Published:** 2026-09-30

**Authors:** Oddny Bjorgvinsdottir, Karin Wuertz-Kozak, Bergthora Sigridur Snorradottir, Thorarinn Gudjonsson, Andrea Heinz, Sigurdur Runar Gudmundsson, Stephen John Ferguson

**Affiliations:** 1 Faculty of Pharmaceutical Sciences, University of Iceland, Hofsvallagata 53, 107 Reykjavik, Iceland; odb9@hi.is (O.B.); bss@hi.is (B.S.S.); 2 Department of Biomedical Engineering, Rochester Institute of Technology (RIT), 160 Lomb Memorial Drive Bldg. 73, Rochester, NY 14623, USA; kwbme@rit.edu; 3 Faculty of Medicine, School of Health Sciences, University of Iceland, 101 Reykjavik, Iceland; tgudons@hi.is; 4 Section of Laboratory Hematology, Landspitali University Hospital, 101 Reykjavik, Iceland; 5 Department of Pharmacy, LEO Foundation Center for Cutaneous Drug Delivery, University of Copenhagen, Universitetsparken 2, 2100 Copenhagen, Denmark; andrea.heinz@sund.ku.dk; 6 Biomedical Center, School of Health Sciences, University of Iceland, 101 Reykjavik, Iceland; srg@hi.is; 7 Institute for Biomechanics, ETH Zurich, Gloriastrasse 37/39, 8092 Zurich, Switzerland

**Keywords:** electrospun fibers, polydimethylsiloxane (PDMS), composite, adhesion

## Abstract

The increasing use of complex medical devices has intensified the need for reliable integration of multiple biocompatible materials. Silicone elastomers are widely used in implantable devices and frequently combined with materials designed to support cell growth. In such hybrid systems, robust interfacial adhesion is essential for mechanical stability and functional performance. In this study, electrospun poly(ε-caprolactone) (PCL) fibers were deposited directly onto two silicone elastomers, RTV 4420 and Sylgard 184, to form mechanically integrated bilayer structures. Adhesion of the electrospun PCL membrane to the silicone substrates was evaluated using a 180° peel test, while fiber morphology and surface topography were characterized to investigate the influence of the substrate material. The mean steady-state adhesive strength measured for the electrospun PCL membrane and RTV 4420 was 4.76 ± 0.05 mN/mm (mean ± SEM), compared with 6.83 ± 1.49 mN/mm for Sylgard 184. Distinct differences in residual bead formation and fiber morphology were observed between fibers collected on polydimethylsiloxane (PDMS) and aluminum foil. Fiber diameter differed significantly when spun onto uncured Sylgard 184 compared with uncured RTV 4420 or aluminum foil. Additionally, high-voltage electrospinning induced transient surface structures in the uncured silicone that were retained in the fiber pattern after voltage removal. Overall, direct electrospinning enabled PCL–silicone adhesion while providing control over fiber morphology, offering a promising approach for developing stable, cell-supporting interfaces in research and medical devices.

## 1. Introduction

The increasing complexity of modern medical devices necessitates the integration of diverse biocompatible materials to achieve the required combination of mechanical, biological, and functional properties. Reliable adhesion between dissimilar materials is essential for maintaining structural integrity and preventing interfacial failure or delamination during long-term use under physiological conditions [1]. This challenge is particularly relevant for systems combining polydimethylsiloxane (PDMS) elastomers with cell-supportive fibrous substrates, where the chemical inertness of PDMS and the delicate structure of fibrous scaffolds can limit conventional approaches to interfacial integration.

PDMS is widely used in biomedical applications because of its elasticity, chemical stability, and biocompatibility [2,3,4,5,6]. Its silicon–oxygen backbone provides flexibility and high gas permeability, while its hydrophobic and chemically inert surface contributes to its stability in demanding environments [7]. Consequently, PDMS has been incorporated into a range of medical devices and biomedical systems, including catheters, prosthetic components, and drug delivery devices [8]. Sylgard 184 and RTV 4420 are two widely used two-part PDMS elastomers with distinct mechanical properties. Sylgard 184 is a well-characterized and mechanically robust elastomer commonly used in microfluidics, lithography, and biomedical research [9,10], whereas RTV 4420 provides a softer and more compliant material suitable for applications requiring greater flexibility. Both materials undergo platinum-catalyzed hydrosilylation, in which vinyl-functionalized PDMS reacts with Si-H-containing crosslinkers to form the crosslinked silicone network [10,11,12,13].

Despite their widespread use, PDMS elastomers present challenges for integration with other biomaterials because of their low surface energy [14], hydrophobicity [15], and chemical inertness [16]. Poly(ε-caprolactone) (PCL), in contrast, is a widely investigated synthetic biomaterial for tissue engineering because of its biocompatibility, biodegradability, mechanical properties, and processability [17,18]. Electrospinning is commonly used to fabricate PCL scaffolds with fibrous architectures that can reproduce important structural features of the extracellular matrix [19,20,21]. These electrospun matrices, can be combined with PDMS to create composite systems that provide both mechanical support and a fibrous environment conducive to cellular functions such as attachment and proliferation [9,22,23]. Depending on their architecture, such fibrous matrices can reproduce features of basement membranes or interstitial extracellular matrices across the nano- to micrometer scale [23,24]. However, integrating delicate electrospun scaffolds with PDMS presents a particular challenge. The small fiber dimensions and porous architecture of electrospun scaffolds make them susceptible to structural deformation during post-fabrication processing. Conventional approaches involving mechanical pressure or elevated temperatures may alter the scaffold morphology, while the use of additional chemical adhesives introduces another material into the system and may complicate fabrication and regulatory considerations. Furthermore, the low surface reactivity of PDMS can limit the formation of strong interfacial bonds with PCL [25,26,27]. Therefore, methods that enable direct integration of electrospun fibers with PDMS while preserving the fibrous architecture are needed.

Previous studies have demonstrated the direct integration of electrospun fibers with PDMS by depositing fibers onto uncured elastomer, allowing the fibers to become embedded within the matrix during curing [27]. This approach has been demonstrated using materials such as poly(vinylidene fluoride-co-hexafluoropropylene) (PVDFhfp), indicating that direct electrospinning onto uncured PDMS can provide an alternative to conventional post-fabrication bonding strategies [27]. However, the applicability of this approach to electrospun PCL fibers, which are of particular interest for biomedical and tissue engineering applications, remains insufficiently investigated. Differences in polymer chemistry, fiber morphology, and mechanical properties may influence fiber deposition and morphology in and on top of the uncured elastomer and the resulting adhesion to the underlying PDMS.

**Figure 1:**
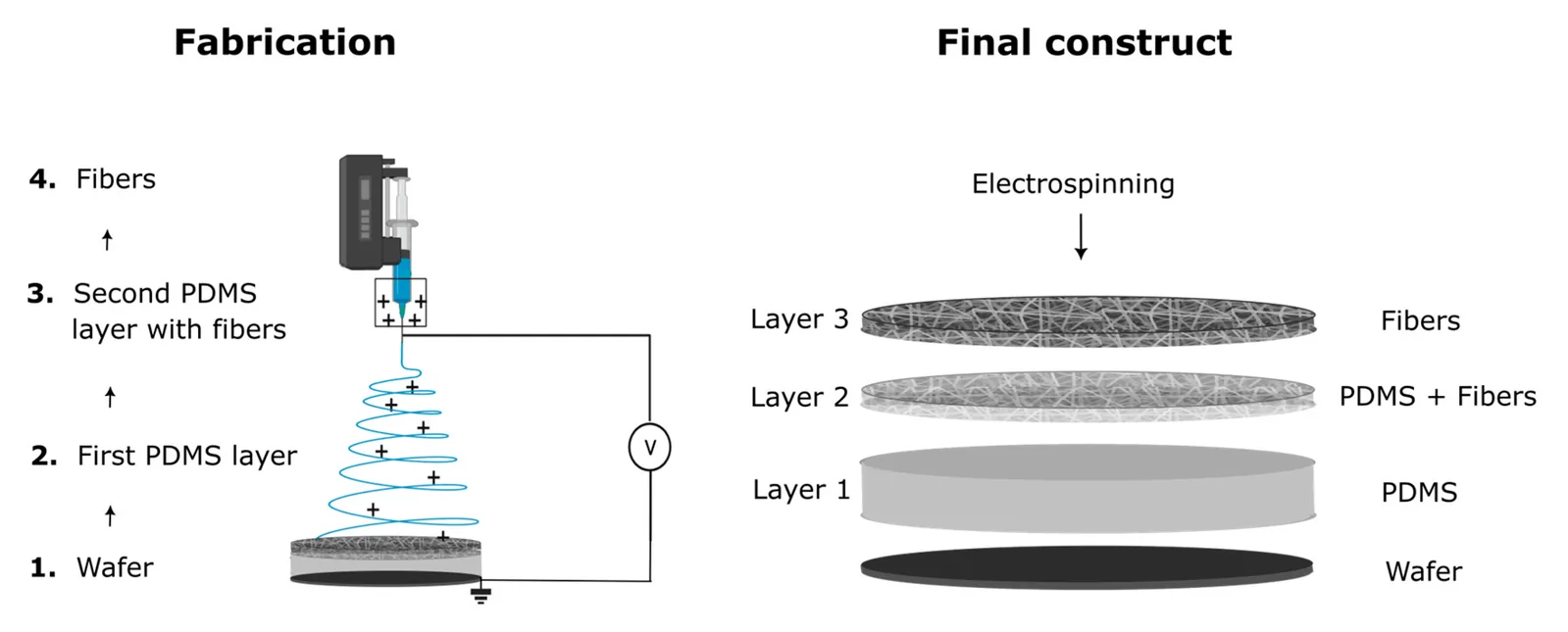
Schematic illustration of the fabrication process and final layered construct. Silicone elastomer layers were applied to a silicon wafer, followed by direct electrospinning of PCL fibers onto the uncured upper silicone layer.

In this study, we investigate the direct electrospinning of PCL fibers onto uncured PDMS as a strategy for integrating electrospun PCL scaffolds with silicone elastomers. The fibers are deposited directly onto the uncured PDMS and become embedded within the elastomer during curing, forming a mechanically integrated fiber–elastomer interface. The fabrication process and the final construct are shown in Figure 1. We evaluate the resulting PCL–PDMS composite and compare its behavior for two commonly used PDMS formulations, RTV 4420 and Sylgard 184. This work extends the direct fiber–PDMS integration approach to PCL and examines how the properties of the elastomer influence the resulting interface. The approach may provide a simple route for fabricating flexible PCL–PDMS composites with potential applications in biomedical devices and tissue engineering constructs.

## 2. Results

### 2.1. Spin Coating Curve for RTV 4420

**Figure 2:**
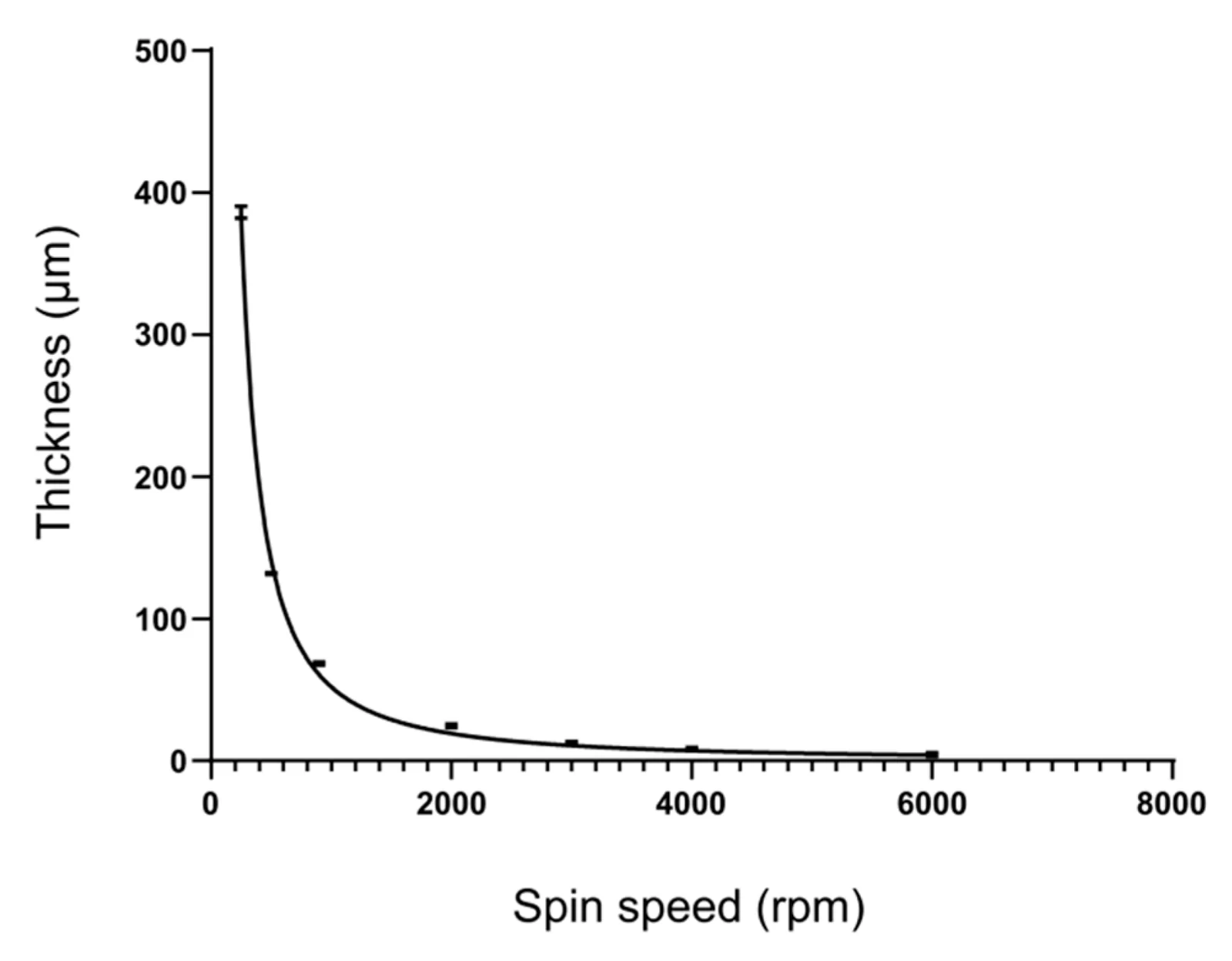
RTV 4420 PDMS thickness as a function of spin speed. Films were spun for 60 s with 20 rpm s^−1^ acceleration. The experimental data were fitted using a power-law relationship, yielding t(ω) = 1.11·10^6^ ω^−1.44^ (t in µm, ω in rpm).

The spin coating curve for RTV 4420 silicone containing 1.5% inhibitor is illustrated in Figure 2. The data were fitted using a power-law model of the form t(ω) = Aω^B^, where t is the film thickness [µm] and ω is the spin speed (rpm). Nonlinear regression yielded best-fit parameters of A = 1,113,448 and B = −1.44. The resulting fit,

t(ω) = 1.11 ∙ 10^6^ *ω*^−1.44^

describes the thickness–spin speed relationship with excellent agreement (R^2^ = 0.9982).

The relationship between the spin speed and the resulting film thickness for Sylgard 184 has been reported previously [28,29]. This relationship has been described as the empirical equation ${t_{\mathrm{Sylgard}184}=49,901f}^{-0.993}$ where t is the film thickness and f the spin speed [29] as well as the theoretical fit t(ω) = 0.23 ω^−1.14^ [28]. The exponent of the power-law fit for RTV 4420 (−1.44) is more negative than that reported for Sylgard 184 (−1.14), indicating a stronger dependence of film thickness on spin for the RTV 4420. To determine the thickness of the silicone layers used in the present study, the upper layers of both RTV 4420 and Sylgard 184 were measured at the spin speed used for fabrication of the composite constructs (4000 rpm). The resulting thicknesses were 8.5 ± 0.6 µm for RTV 4420 and 7.02 ± 0.6 µm for Sylgard 184. These measurements provide the actual thicknesses of the silicone layers used in the subsequent experiments.

### 2.2. Substrate Characteristics Across Underlying Base Materials

**Figure 3:**
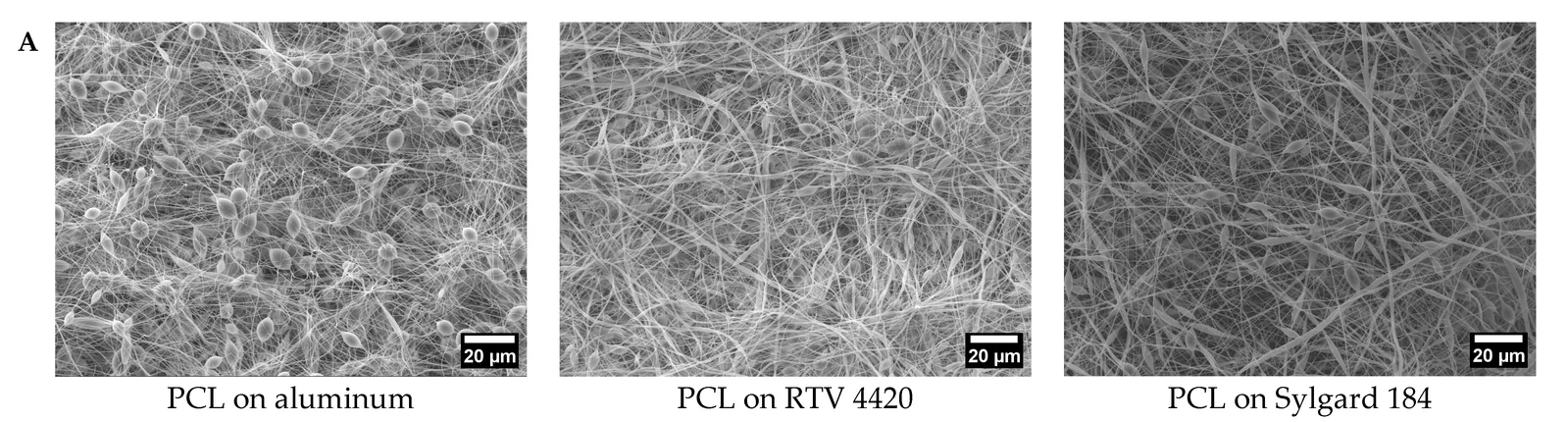

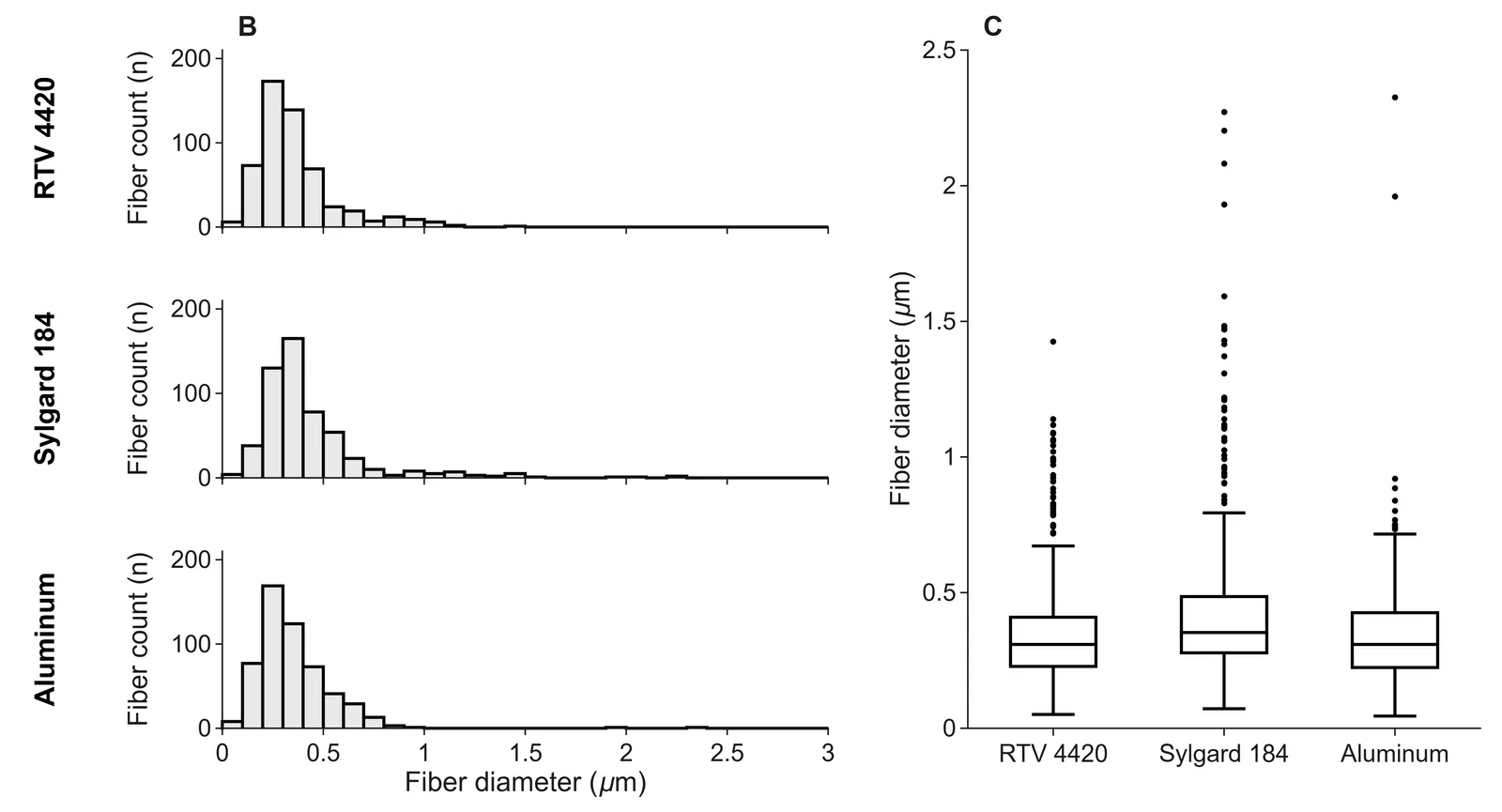
PCL fibers spun on aluminum foil and on the uncured RTV 4420 and Sylgard 184 elastomers. Scanning electron microscopy images show the morphology of PCL fibers (**A**) spun on aluminum foil (**left**), uncured RTV 4420 (**middle**) and uncured Sylgard 184 (**right**). Histograms (**B**) and box plots (**C**) showing the fiber diameter distribution. Fiber diameter of PCL spun on Sylgard 184 was significantly larger (*p* < 0.05).

Figure 3 shows the substrate characteristics across the underlying base materials. The three conditions correspond to PCL fibers electrospun onto aluminum foil, uncured RTV 4420 and uncured Sylgard 184, respectively. Representative scanning electron microscopy images of the fiber-covered surfaces are shown in Figure 3A. Qualitative differences in bead formation were observed for fibers deposited on the uncured silicone substrates compared with aluminum foil, with beads on PDMS showing a more elongated morphology. The distribution of fiber diameters for each substrate is presented as histograms in Figure 3B and as box plots in Figure 3C. The fiber diameter of PCL spun on Sylgard 184 (0.427 µm) differed from that of the other two substrates (0.346 µm on aluminum foil and 0.355 µm on RTV 4420), according to Kruskal–Wallis one-way ANOVA (*p* < 0.05) and Dwass–Steel–Critchlow–Fligner pairwise comparison. QQ plots and the Shapiro–Wilk test demonstrated that the fiber diameters were not normally distributed (Supplementary Material, Figure S1).

### 2.3. Adhesion Between PDMS and PCL

**Figure 4:**
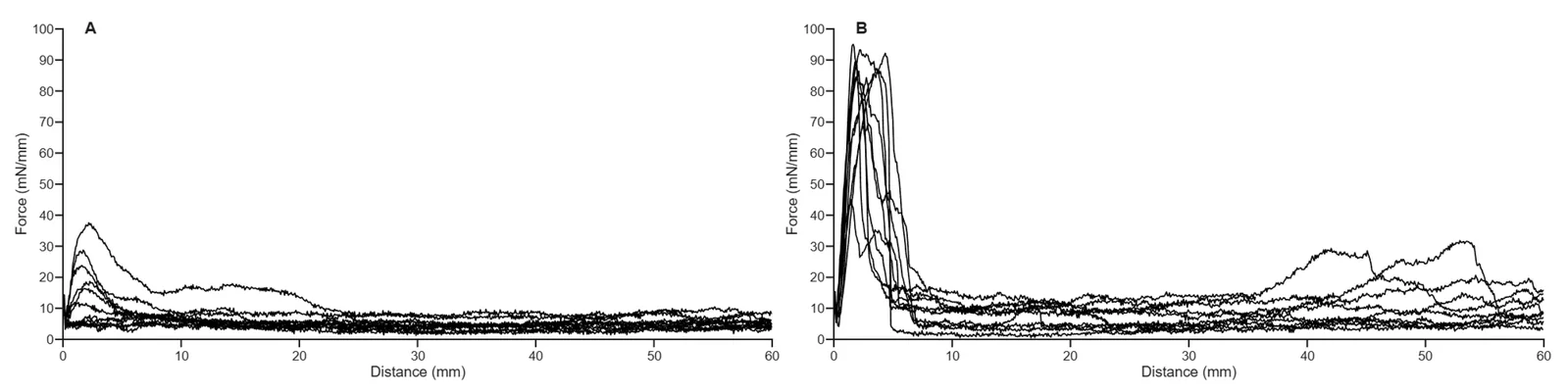
The adhesive strength between RTV 4420 and PCL fibers (**A**) and Sylgard 184 and PCL fibers (**B**). The mean adhesive strength when PCL fibers were peeled off RTV 4420 and Sylgard 184 were 5.63 ± 0.73 mN/mm and 11.97 ± 0.51 mN/mm (mean ± SEM), respectively. The mean adhesive strength differed by 6.34 mN/mm.

Figure 4 presents the adhesive strength between electrospun PCL fibers and RTV 4420 silicone (Figure 4A), and electrospun PCL fibers and Sylgard 184 silicone (Figure 4B). The two materials did not part at the elastomer–fiber interface, but separated in layers within the fibrous substrate. The mean adhesive strength over the 60 mm peel off region was 5.63 ± 0.73 mN/mm (mean ± SEM) for fibers on RTV 4420, but 11.97 ± 0.51 mN/mm (mean ± SEM) for fibers on Sylgard 184. The mean adhesive strength differed by 6.34 mN/mm. The overall mean adhesive strength across both elastomers was 8.80 ± 0.62 mN/mm (mean ± SEM).

The steady-state adhesive strength was calculated as the average force measured after the initial detachment region. It was calculated over a displacement range of 10–30 mm for Sylgard 184 and 30–60 mm for RTV 4420. For the steady-state region, the two individual RTV batches yielded mean adhesive strengths of 4.71 and 4.81 mN/mm, respectively. The two individual Sylgard batches yielded mean adhesive strengths of 5.34 and 8.32 mN/mm, respectively. The mean steady-state adhesive strength for RTV was 4.76 ± 0.1 mN/mm and 6.83 ± 1.5 mN/mm for Sylgard (mean ± SEM). The steady state adhesive strength differed by 2.07 mN/mm.

**Figure 5:**
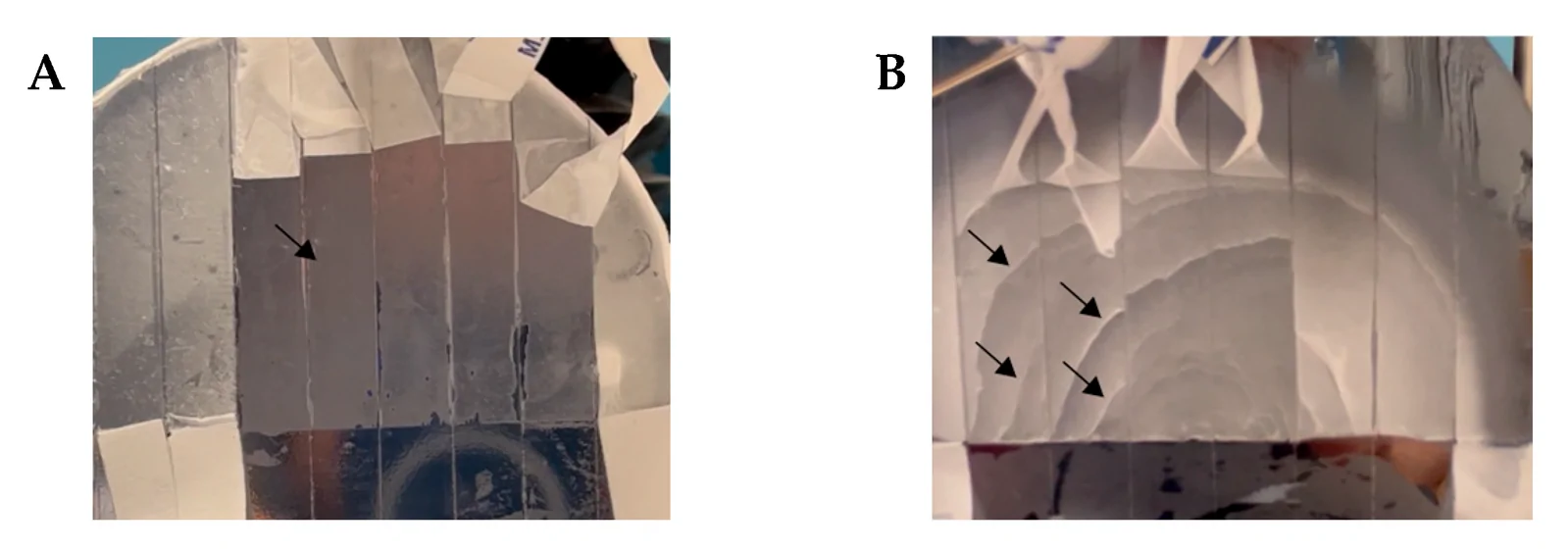
Distribution of PCL on the collector after the peel test. The deposition during electrospinning resulted in a layered membranes. The fibers spun on RTV 4420 showed uniform fiber distribution (**A**) but fibers spun onto Sylgard 184 were deposited in circular areas that gradually increase in size. The arrows point at the intersection of these layers (**B**).

Fibers on RTV 4420 were evenly spread throughout the whole wafer (Figure 5A) but on Sylgard 184 fibers accumulated in gradually increasing circles (Figure 5B). This heterogeneous accumulation of fibers, which resulted in layering, was clearly evident after the fibrous layer had been peeled off (Figure 5).

### 2.4. Tensile Testing

**Figure 6:**
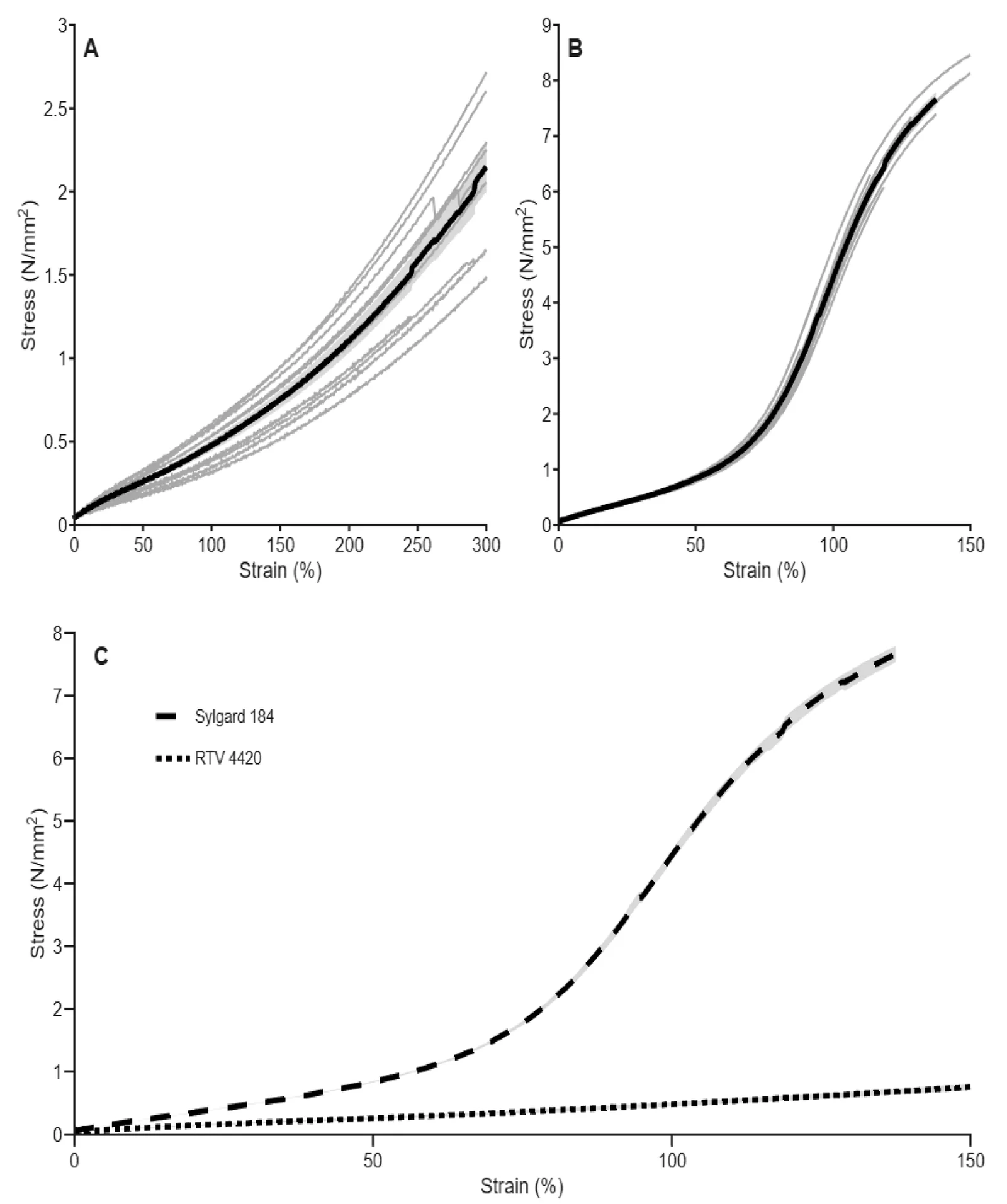
Mechanical characterization of silicone elastomers. Stress–strain curves of (**A**) RTV 4420 and (**B**) Sylgard 184 showing individual measurements (grey lines), mean curves (black line), and SEM (shaded area). (**C**) Comparison of the mean stress–strain response of RTV 4420 and Sylgard 184 within the overlapping strain range (0–150% strain).

Figure 6 shows stress–strain curves of RTV 4420 and Sylgard 184 elastomers. RTV 4420 specimens were evaluated up to 300% strain, whereas Sylgard 184 specimens were evaluated up to 150% strain because of earlier specimen failure. Mean ± SEM are shown only where at least four specimens contributed. The Sylgard 184 silicone elastomer (Figure 6B) exhibited greater and more uniform stiffness than RTV 4420 (Figure 6A), as indicated by the steeper initial slope of the stress–strain curve and the higher values at equivalent strains. RTV 4420 was softer than Sylgard 184 and exhibited greater batch-to-batch variability, resulting in less uniform stress–strain curves. Figure 6C directly compares the mechanical response of RTV 4420 and Sylgard 184 within the overlapping strain range (0–150%). Mean stress–strain curves are shown with the standard error of the mean (SEM), indicated by shaded regions. For comparison of stiffness, the secant modulus was calculated at 10%, 20% and 50% strain. For RTV, the secant moduli at these strain values were 0.97 ± 0.11, 0.68 ± 0.10, 0.50 ± 0.07 MPa (mean ± SEM), and 2.14 ± 0.01, 1.75 ± 0.02, 1.67 ± 0.04 MPa (mean ± SEM) for Sylgard 184.

The energy absorbed up to 50% strain was quantified by calculating the area under the stress–strain curve (AUC). The AUC up to 50% strain was 0.4 ± 0.1 N/mm^2^ (mean ± SEM) for RTV 4420 and 1.7 ± 0.1 N/mm^2^ (mean ± SEM) for Sylgard 184.

### 2.5. Fiber Anchoring into PDMS

**Figure 7:**
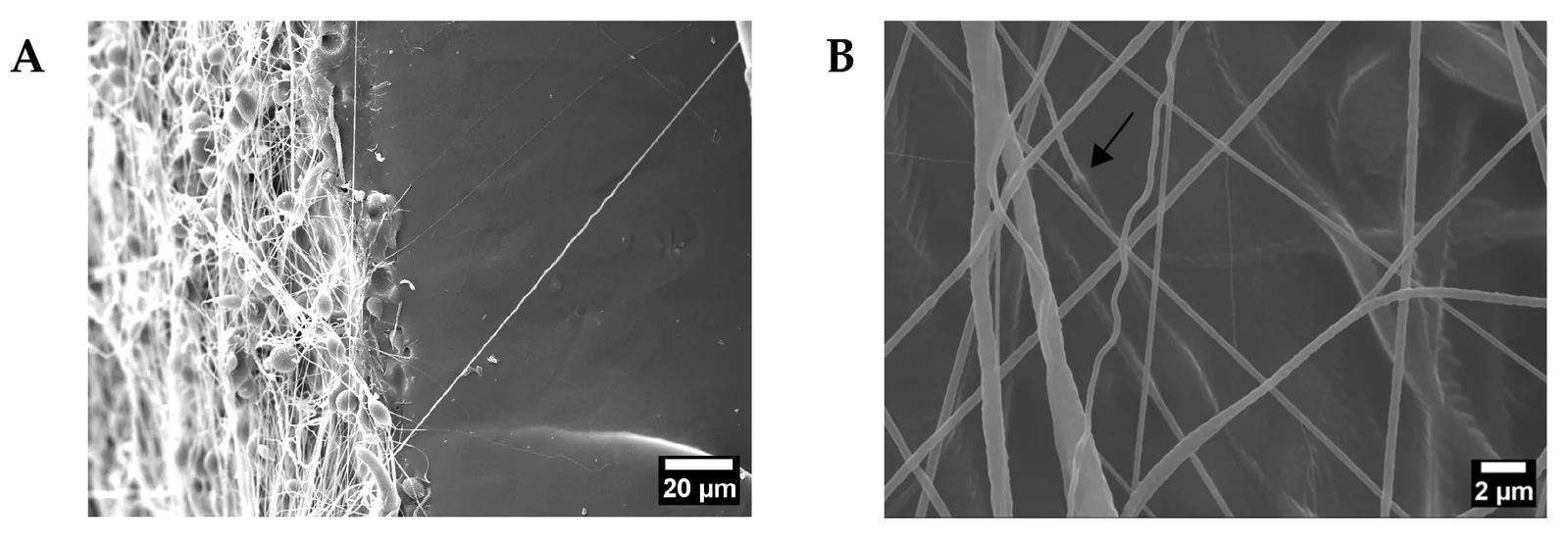
Cross-sectional and surface scanning electron micrographs demonstrating fiber embedding within the PDMS matrix. The cross section shows the distinct layers of the film, the elastomeric films to the right and the fibrous layer on the left (**A**), while the surface image (**B**) shows fibers protruding from and traversing the PDMS surface. The arrow shows an example of fiber traversing the elastomer.

Electrospinning facilitates fiber embedding and mechanical interlocking within the uncured PDMS layer, as confirmed by cross-sectional and surface scanning electron micrographs in Figure 7. Figure 7A show the cross section of the elastomer film with the fibrous layer on top. Figure 7B shows the elastomeric surface with fibers starting to protrude through the surface.

### 2.6. Structured PDMS Surface Under High Voltage and Resulting Fibrous Spin Pattern

**Figure 8:**
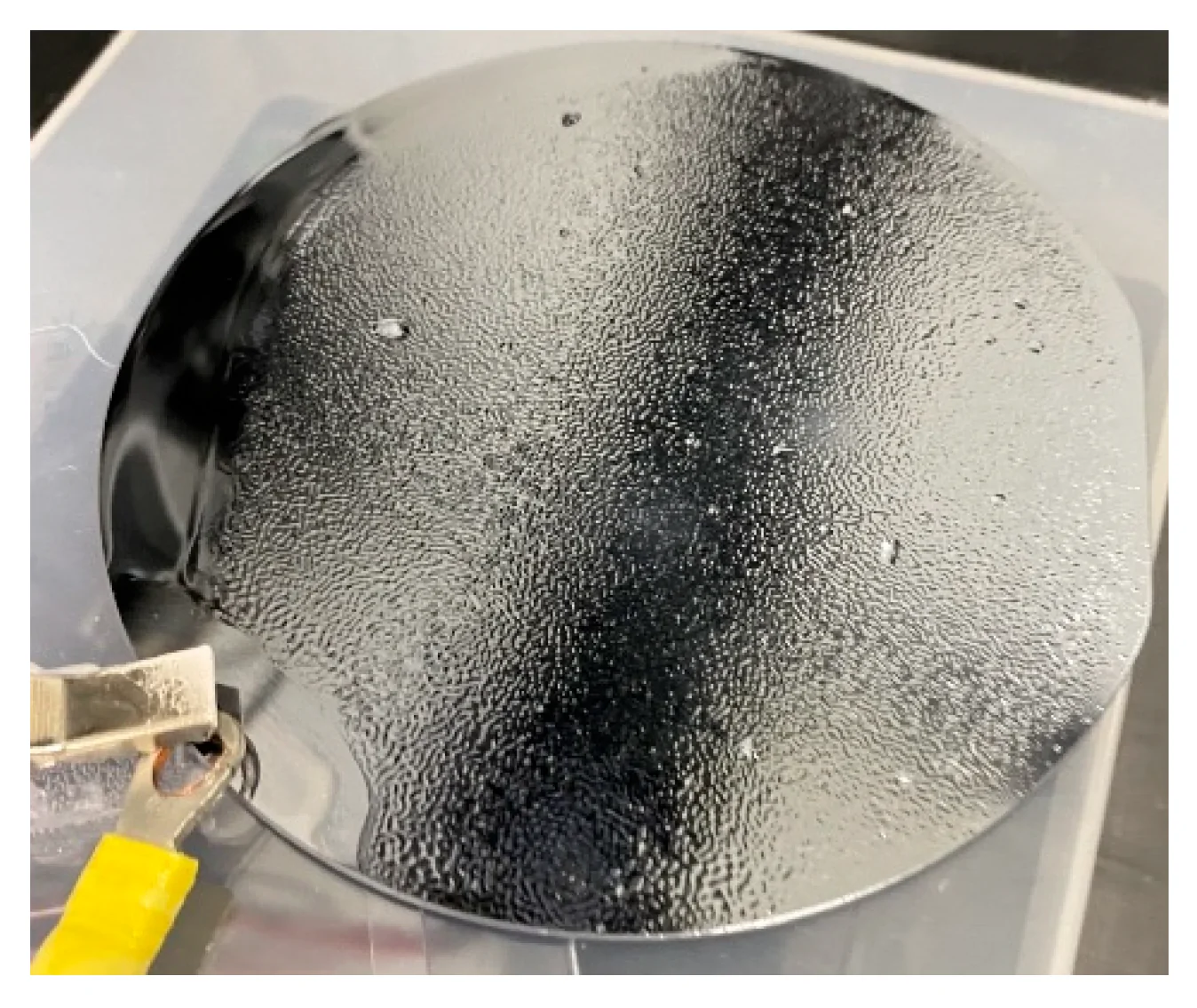
Uncured PDMS placed under high voltage. The surface became unstable after exceeding a critical voltage level.

When an uncured layer of PDMS was put under high voltage, the initially flat surface became unstable above a critical voltage, forming hills and valleys. Patterning first appeared at 13 kV and increased in height and area with increasing PDMS layer thickness (Figure 8). The observations were reproducible across two independently prepared batches.

**Figure 9:**
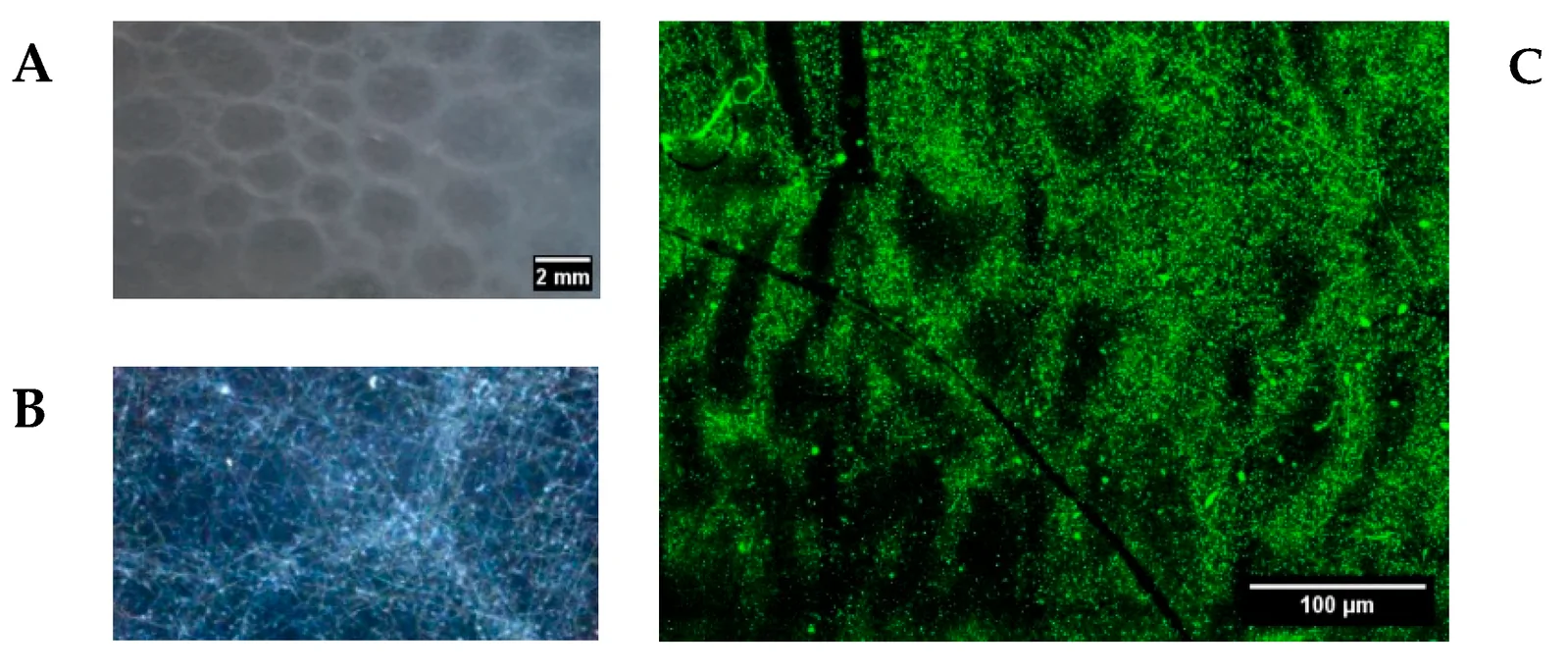
(**A**) Photograph image of fibers forming a circular pattern when spun onto uncured silicone elastomer under high voltage. (**B**) Magnification of the resulting fiber pattern, microscopy image (~100× magnification) and (**C**) confocal image of the resulting fiber pattern.

When PCL fibers were spun onto the structure, they merged with the PDMS and formed a pattern. Figure 9 shows photograph, microscopy and confocal images of the resulting fiber pattern.

## 3. Discussion

A spin coating curve showing the dependence of PDMS thickness on spin rate was established for RTV 4420 with 1.5% inhibitor. Similar spin coating curves for Sylgard 184 have previously been reported by Zhang et al. [28] and Schneider et al. [29]. For a polymer solution under negligible solvent evaporation conditions, the classical Emslie–Bonner–Peck spin coating model predicts that the thickness of the spin-coated film is inversely proportional to the spin speed, (h ∝ ω^−1^) [30]. This relationship assumes a Newtonian liquid with constant viscosity and negligible evaporation. In silicone elastomers such as PDMS, however, viscosity may increase during spinning due to the onset of curing, and non-Newtonian behavior can occur at high shear rates [31]. These effects may lead to deviations from the ideal h ∝ ω^−1^ scaling, as reported for Sylgard 184 by Zhang et al. [28] and Schneider et al. [29], with scaling exponents of approximately −0.99 and −1.14, respectively. The more negative scaling exponent observed for RTV 4420 (h ∝ ω^−1.44^) may indicate a somewhat stronger dependence of film thickness on spin speed than that reported for Sylgard 184. Nevertheless, the spin coating behavior was sufficiently similar to allow spin coating parameters established for Sylgard 184 to serve as a useful basis for preparing RTV 4420 films. At 4000 rpm, the measured film thicknesses were 8.5 ± 0.6 µm for RTV 4420 and 7.02 ± 0.6 µm for Sylgard 184. The higher thickness measured for RTV 4420 at this spin speed demonstrates that the difference in scaling exponents does not directly translate into a lower absolute film thickness. The observed difference in scaling may reflect differences in the rheological behavior of the two elastomer formulations during spinning, however, this was not directly investigated in this study.

To increase the pot life of RTV 4420, an inhibitor was used to slow down the cross-linking reaction by temporarily suppressing the platinum catalyst. In the present work, a corresponding spin coating curve for RTV 4420 with 1.5% inhibitor is presented. The addition of an inhibitor is necessary for the electrospinning process when producing thin fibers or when the objective is to obtain a thick spun layer, for example, for applications such as three-dimensional scaffolding.

The bead-on-string morphology of the spun membranes are in general not desirable for cellular scaffolds, as uniform fibers better replicate the native extracellular matrix and beads disrupt fiber architecture. To create more uniform membrane with smooth fibers, the conductivity of the spinning solution can be increased, for example, by the addition of salts [32,33]. Increased solution conductivity increases charge density in the jet, and therefore enhances stretching forces during electrospinning. In some cases, however, a bead-on-string morphology is desired. These applications include certain types of drug delivery, catalytic supports and textured surfaces to increase surface roughness [34,35,36]. In this study, the resulting fibrous PCL layer spun on uncured silicone and aluminum foil showed a bead-on-string morphology, but with some structural differences. The bead shape was slightly affected, with beads showing a rounder morphology on the aluminum foil compared to beads spun on uncured PDMS. As the electrospinning parameters were kept constant for all three materials at the collector, the differences are likely related to differences in electrical conductivity, surface energy, and charge dissipation at the collector surface, which influence the final stretching and solidification of the electrospinning jet upon deposition. Compared to aluminum foil, uncured silicone substrates exhibit low surface energy [37,38], viscoelastic compliance [39,40], solvent uptake [41], and low electrical conductivity [42,43], all of which can prolong the liquid state of deposited fibers. This can lead to continued jet stretching and the formation of elongated beads rather than compact hemispherical droplets as seen on the aluminum foil. The broader distribution of the fiber diameter of PCL spun on Sylgard 184 compared to PCL spun on either RTV 4420 or aluminum foil shows that the underlying material can also affect the fiber topography. Despite both Sylgard 184 and RTV 4420 being silicone elastomers, a subtle difference in material chemistry and electric behavior seems to be sufficient to significantly alter the fiber diameter distribution in this way. The influencing factors could be their catalyst chemistry, dielectric constant, ionic mobility and/or interfacial charge transport. As many of these physical properties have not been characterized for RTV 4420, the mechanisms by which specific material properties influence fiber diameter, its distribution, the deposition pattern, and adhesion, remain hypothetical. Differences in electrical conductivity between the elastomer substrates may, however, affect charge dissipation and consequently influence the resulting electrospun construct.

Adhesion testing quantified the force required to delaminate the electrospun fibrous layer from the PDMS substrates. For both material types (PCL on Sylgard 184 and PCL on RTV 4420), failure did not occur at the PDMS–fiber interface but rather within the fibrous membrane, specifically between fiber layers on top of the PDMS–fiber interface. This cohesive failure indicates that fiber anchoring to the PDMS substrate is stronger than the interlayer adhesion within the fibrous network [44]. Several studies have investigated the adhesion of electrospun fibers to base substrates. Fibers deposited directly onto solid substrates typically exhibit relatively weak adhesion. This adhesion generally arises from van der Waals interactions associated with the fibrous structure of electrospun mats. Electrospun meshes possess a high surface-area-to-volume ratio and a large number of polymer chains exposed at the fiber surface, which can promote intermolecular interactions [45,46,47]. In addition, residual electrostatic charges generated during the electrospinning process may contribute to the adhesion between the fibers and the substrate [46]. Hellert et al. looked at the adhesion of electrospun fibers on conductive and insolating foil substrate without an adhesive [48]. Even without introducing an adhesive, there is considerable variation in the adhesive strength, ranging from 0 to 8 mN/mm depending on substrate [48]. The lowest adhesive strength was on a non-conductive substrate but the highest on carbon with polystyrene sulfonate [48]. The adhesive strength between electrospun layers show slightly stronger adhesion (or up to 11 mN/mm) [49]. Electrospun fibers on a melt-electrowritten PCL grid show lower adhesive strength (or 3–4 mN/mm), probably due to less contact area of the two substrates [50]. Several methods have been developed to increase the adhesion of electrospun fibers to an elastomer; these include chemical methods to modify the surface roughness, surface chemistry or polymer chain interactions [45]. Of these methods, the modification of the surface chemistry that causes polymer chain entanglement has been the most effective, increasing the adhesive strength up to 44 mN/mm [45]. Other approaches for improving the adhesion of electrospun fibers to non-elastomeric substrates have involved solvent treatment or thermal treatment using a hot-melt adhesive [51,52]. For example, Khavarzamini and Merati treated nonwoven polyester substrates with dimethylformamide (DMF)/water solutions before electrospinning PAN nanofibers onto the treated substrate, achieving a maximum peeling force of 9.61 cN under the optimal treatment conditions [52]. Amini and Gharehaghaji, in contrast, incorporated poly(vinyl acetate) (PVAc) nanofibers as a hot-melt adhesive into electrospun nylon 66 mats deposited on woven polyester fabric and subsequently applied thermal treatment to improve adhesion [51].

A limitation of the adhesion experiments is the small number of independently prepared batches (n = 2 for each elastomer). The average adhesive strength of 8.8 ± 0.6 mN/mm (n = 4, mean ± SEM) was calculated from four specimens, with two specimens from each elastomer. The average adhesive strength presented in this study is comparable to previously reported adhesion between electrospun fibers to solid substrates [49,50,51] but lower than that reported for PVDFhfp fibers electrospun onto elastomers (40–170 mN/mm) [27]. This indicates that substantially higher adhesive strengths may be achievable if failures within the layered PCL fiber membrane can be overcome. Such an improvement could increase the mechanical integrity and robustness of the resulting fiber–elastomer construct. 

Notably, the spatial distribution of fibers differed markedly between the two PDMS formulations. On RTV 4420, fibers were deposited relatively uniformly across the entire wafer surface, whereas on Sylgard 184, fibers accumulated in concentric rings centered on the wafer, with progressively increasing surface coverage toward the outer regions. This non-uniform deposition pattern was apparent during electrospinning and remained clearly visible after peel-off during adhesion testing. As the nanofiber layer homogeneity on the collector depends on the uniformity of the electric field [53], the difference in deposition pattern is likely a consequence of substrate properties during deposition. According to Skrivanek et al. [53], the collector form and shape can influence this uniformity so modification of the collector could possibly solve these differences of fiber deposition. It remains unclear whether the differences in spatial fiber distribution contributed to the differences in adhesive strength measured between the electrospun fibers and the two silicone elastomers, RTV 4420 and Sylgard 184. However, it is likely that differences in the chemical composition and electrical response of the two elastomers influenced the adhesion, either directly through interfacial interactions or indirectly by affecting fiber morphology and topography. Variations in fiber diameter and arrangement can influence the real contact area and mechanical interlocking between the electrospun membrane and the substrate, with larger fibers generally reported to exhibit stronger adhesion in electrospun systems [54]. Furthermore, differences in cross-linking behavior during the pot-life window may affect the electrical properties of the elastomers, including charge dissipation and charge accumulation at the fiber–elastomer interface and through the underlying silicon wafer. These changes in the electrostatic environment during electrospinning may consequently influence fiber deposition and the resulting adhesion strength.

When a spin-coated layer of PDMS is placed under voltage that exceeds a critical value, the surface becomes unstable. This electromechanical instability causes a pattern of hills and valleys to emerge. For a free-standing PDMS layer, the film would decrease in thickness; however, as the PDMS in this case is bound to the wafer, the lateral expansion is constrained so the vertical deformation dominates. Small bumps grow taller and nearby regions sink, which leads to wrinkles or surface corrugation [55]. The dominant effect of this electromechanical instability is Maxwell stress, also known as Maxwell pressure [56]. When a voltage between compliant electrodes is applied and pressure arises, the elastomer compresses while it expands in the plane. In 2008, Zhou et al. showed the Maxwell stress as the source of surface instability in PDMS membranes [57]. Electrospinning onto structured PDMS creates a patterned fiber deposition. Fibers accumulate in the valleys and even after the voltage is turned off and the surface structure of the PDMS returns to a flat film, the fibers remain patterned. This formation of a network could be useful in the reinforcement of PDMS for various applications.

One of the most common methods for chemically bonding materials to PDMS is silanization [58]. Because PDMS is chemically inert and hydrophobic, direct bonding to polymers is typically weak. Silanization introduces reactive silane groups that can form covalent bonds with the PDMS surface while providing functional groups that bond to the polymer, thereby creating a molecular bridge between the two materials [58,59]. However, this approach requires surface activation, commonly by plasma treatment or similar methods, and involves the use of silanes, some of which are moisture sensitive and toxic [30,60]. The process is therefore multistep and more complex, although it results in a thin interfacial layer on the nanometer scale [58,61]. Silane-based surface functionalization has also been used to promote the adhesion of electrospun fiber networks to PDMS, for example, through methacrylation of PDMS surfaces using 3-(trimethoxysilyl)propyl methacrylate [62]. In contrast, the present approach enables direct integration of electrospun PCL fibers with uncured silicone elastomers without an additional surface functionalization or adhesive layer.

The main advantage of the embedding method presented here is that no other materials are needed to bind the fibrous layer to PDMS. Also, the delicate surface structure remains intact since no pressure or handling is needed for this type of adhesion. Rhyou et al. also used uncured PDMS to bind fibers to a substrate but silver nanowires and compression was used additionally to achieve the adhesion [63]. A method with no additional materials and no compression can be especially useful in the fabrication of porous scaffolds where cell infiltration is of great importance. The method can be applied to other PDMSs if the pot life is long enough for the electrospun fibers to emerge from the uncured layer and accumulate on top. Larger-diameter fibers (≥1 µm) accumulate on the collector over relatively short electrospinning times; however, producing fibers with diameters comparable to those of fine native extracellular matrices [23], such as the human cornea (46 ± 16 nm) [64] or human dermal collagen (50–150 nm) [65,66], typically requires substantially longer spinning durations [67]. For the fabrication of small diameter fibers, an inhibitor can be added to the PDMS to prolong its pot life [68,69]. This allows the embedding of fibers before curing takes place. The spin time also depends on the thickness of the uncured material as first the fibers need to accumulate within that layer before reaching the surface. As the fiber distribution on the surface can be different between PDMS types, a moving spinneret might help with creating a uniform layer. Methods that eliminate layering can furthermore improve the integrity of the combined structure.

As PDMS has low conductivity, it is important to ensure good conductivity of the collector. In our case, silicon wafers were used as a base for the spin coating and used as a collector during the electrospinning. Doped (1–20 Ω·cm) wafers were chosen due to their increased conductivity. It is important to note that the electric circuit for electrospinning needs to be complete; if the wafer used is poorly conducting, a conductive material needs to be added to drive the spinning process. This could be done by introducing any conductive material, such as metal particles, to the PDMS layer.

## 4. Materials and Methods

### 4.1. Materials

Two silicone elastomers were used as base layers for electrospinning medical-grade RTV 4420 silicone elastomer (Elkem Silicones, Oslo, Norway) with additional 1.5 wt% inhibitor (COP Chemie, Saint-Nazaire-en-Royans, France) and Sylgard 184 PDMS with a 10:1 base-to-curing-agent ratio (Dow Corning, Midland, MI, USA). The polymer Polycaprolactone (PCL; average Mn 80,000 g mol^−1^, Sigma-Aldrich, Darmstadt, Germany) was used for electrospinning. The PCL was dissolved in ethanol (96% purity) and chloroform (99.2%, stabilized with ~0.6% ethanol) in a 1:6 (*v*/*v*) ratio mixture, both obtained from VWR Chemicals (Søborg, Denmark).

### 4.2. Spin Thickness Curve

PDMS membranes were produced by spin coating. The relationship between the spin coating speed and resulting membrane thickness was evaluated. A total of 2 g of Silbione RTV 4420 (Elkem, Oslo, Norway) containing 1.5% tetramethyltetravinylcyclotetrasiloxane curing inhibitor (COP Chemie, France) was dispensed onto a 4-inch silicon wafer (WaferPro, San Jose, CA, USA). The samples were spin-coated for 60 s using an acceleration of 20 rpm s^−1^ (Spin-150, SPS Europe, Putten, The Netherlands). Spinning speeds of 250, 500, 600, 900, 2000, 3000 and 4000 rpm were applied. After curing, the thickness of each membrane was measured using a DektakXT stylus profilometer (Bruker, Billerica, MA, USA). Nonlinear regression was used to fit the spin coating data to a power-law model t(ω) = Aω^B^, where t is the thickness and ω is the spinning speed. Nonlinear least-squares regression was performed in GraphPad Prism 10.6.1 (GraphPad software, San Diego, CA, USA) using the Levenberg–Marquardt algorithm.

### 4.3. Membrane Fabrication

Two PDMS silicones, Silbione RTV 4420 and Sylgard 184, were fabricated identically. Two separate layers of silicone were spin-coated. First, 2 g of silicone was spin-coated at 900 rpm onto a 4-inch silicon wafer (W000730, Inseto, Andover, UK; boron-doped, resistivity 1–20 Ω·cm) with an acceleration rate of 20 rpm s^−1^. Then, the spin-coated layer was cured at 60 °C for 1 h. After curing, a second layer of silicone was spin-coated at 4000 rpm on top and placed immediately in an electrospinning instrument LE-50 (Bioinicia-Fluidnatek, Valencia, Spain). The wafer was placed horizontally in a vertical spinning setup. The PCL solution (average Mw = 80,000 g mol^−1^, Sigma-Aldrich, Darmstadt, Germany), composed of 5 wt% PCL in a 1:6 (*v*/*v*) mixture of ethanol and chloroform, was spun onto the uncured silicone layer for 4 h. The electrospinning process was optimized to obtain the smoothest fiber production and minimum disturbance during deposition. The voltage was set to 7.5 kV with a tip-to-collector distance of 16 cm. The spinning solution was pumped out of a 5 mL syringe with an 18 G needle at a feed rate of 200 µL/h. The spinning temperature and relative humidity were 20 °C and 40%, respectively. After electrospinning, the membrane was cured at room temperature for a minimum of one week.

### 4.4. Adhesive Strength

The adhesive strength between the two materials was quantified using a TA.XTplusC texture analyzer (Stable Micro Systems, Surrey, UK) fitted with a 50 N load cell and a 180° peel test rig. The bonded substrate was secured in the lower clamp of the peel rig while the free end of the sample was clamped in the upper grip, ensuring alignment with the peel direction. After mounting the specimen, the probe position was zeroed and a tare force of 49 mN was applied to define the start of the measurement. Peel tests were performed at a constant speed of 5 mm/s (300 mm/min) while maintaining a peel angle of 180°. Force–distance data were acquired at a sampling rate of 500 points per second (pps) using Exponent software (Stable Micro Systems, Exponent Connect, v8.1.11.0). Peel force was evaluated using two approaches: (i) the average force over the full 60 mm peel distance and (ii) the average force within the steady-state peeling region. The steady-state region corresponded to 10–30 mm displacement for Sylgard 184 and 30–60 mm for RTV 4420. In both cases, the measured force was normalized to the specimen width (10 mm) and reported as adhesive strength (mN/mm). Adhesion testing was performed on two batches with five technical replicates per batch.

### 4.5. Tensile Testing

The mechanical properties of the silicone elastomers RTV 4420 and Sylgard 184 were measured using a TA.XTplusC texture analyzer (Stable Micro Systems, Surrey, UK) equipped with a 50 N load cell and a tensile grip fixture. Rectangular specimens were cut to a width of 10 mm and clamped with an initial gauge length of 25 mm between grips. To establish a consistent starting position, a tare force of 49 mN was applied to remove slack, after which the test commenced. Samples were elongated at a crosshead speed of 2 mm/s for 75 mm. Force–distance data were recorded at 500 points per second using Exponent software (Stable Micro Systems). Each test was performed at minimum in quadruplicate (n = 4) to assess repeatability. Mean stress–strain curves were calculated by linear interpolation of individual specimens onto a common 0.5% strain grid. To enable comparison over a common strain interval, the energy absorbed up to 50% strain was quantified by calculating the area under the stress–strain curve (AUC). One specimen was excluded from the analysis because it failed at the grips before reaching 50% strain. As this premature failure was attributed to the test setup rather than the material itself, the resulting incomplete stress–strain curve was not included in the analysis.

### 4.6. Voltage-Induced Surface Patterning

A thin layer of PDMS was spin-coated onto a silicon wafer and placed horizontally in a Fluidnatek electrospinning system (Bioinicia–Fluidnatek, Valencia, Spain). A voltage of 16 kV was applied with a tip-to-collector distance of 16 cm. Subsequently, 5 wt% PCL solution in a 1:6 (*v*/*v*) mixture of ethanol and chloroform was electrospun onto the patterned PDMS surface. The spinning took place at 45% RH and 20 °C. An 18 G needle was used and a feed rate of 200 µL/h. After fiber deposition, the PDMS substrate was allowed to cure at room temperature. The resulting fiber-coated surfaces were imaged using a digital camera (Nikon, Tokyo, Japan), optical microscope (Sulang Technology, Shenzhen, China), and confocal laser scanning microscopy (FV4000; Olympus, Tokyo, Japan).

### 4.7. Scanning Electron Microscopy

Fibers spun onto uncured RTV 4420, uncured Sylgard 184, and on aluminum foil were imaged by scanning electron microscopy. Samples were gold-coated for 40 s, yielding 12.5 nm coating thickness (Agar sputter coater and Agar thickness monitor, Agar Scientific, Stansted, UK). The samples were imaged using a JSM-IT 800 scanning electron microscope (JEOL, Tokyo, Japan). The membranes were fabricated in duplicates. Three samples were taken from each membrane and three images taken per sample. The fiber diameter was measured using ImageJ software (v1.54p, National Institutes of Health, Bethesda, MD, USA) by taking 30 measurements from the top layers of each image, for a total 540 measurements per substrate type [70]. The resulting data were processed and analyzed in MATLAB (R2026a; MathWorks, Natick, MA, USA) and jamovi (v.2.7.18; The jamovi Project Pty Ltd., Sydney, Australia).

### 4.8. Confocal Laser Scanning Microscopy

The patterned PCL fibers in Sylgard 184 silicone were imaged using a FluoView FV4000 confocal laser scanning microscope (Olympus, Tokyo, Japan) to obtain high-resolution fluorescent micrographs. The samples were placed on a glass slide and imaged without additional staining or coating. Excitation was performed with a 480 nm laser, and fluorescence emission was collected using 520 nm spectral detection. A pinhole size of 126 µm was used. Images were acquired using a 10× objective lens. To generate an overview of the sample patterning, the individual images were captured in a 5 × 5 tile scan configuration with automated stage movement and overlap correction. The resulting stitched composite image was assembled automatically using the FluoView software (cellSens FV v3.2.1.85; Evident Corporation, Tokyo, Japan) to provide a seamless representation of the fiber network and its patterning. The image analysis was performed using cellSens Dimension Desktop software (v4.3.1, Olympus Corporation, Tokyo, Japan).

### 4.9. Microscopy Images and Photographs

Patterned fibers in silicone were imaged with a Nikon D7000 digital camera (Nikon, Tokyo, Japan) using Sigma 70 mm F2.8 DG MACRO lens (Sigma, Kawasaki, Japan) and a portable microscope (Sulang Technology, Shenzhen, China).

### 4.10. Statistics

Jamovi was used assess the normality of fiber diameter data and to perform statistical analyses comparing fibers spun on different base materials; uncured RTV 4420, uncured Sylgard 184 and aluminum foil [71]. Normality was assessed using Q-Q plots and the Shapiro–Wilk test. The Kruskal–Wallis test, a nonparametric analog of one-way ANOVA, was used to assess differences in fiber diameter among groups. When significant differences were detected, the Dwass–Steel–Critchlow–Fligner post hoc test was used for pairwise comparisons. Box plots were used to visualize the distribution of fiber diameters [71,72]. MATLAB was used to plot histograms and box plots of the fiber diameters, the stress–strain behavior, and the adhesive strength between fibers and PDMS.

## 5. Conclusions

This study demonstrates that direct electrospinning onto uncured silicone elastomers provides an effective and adhesive-free strategy for integrating electrospun fiber membranes with PDMS-based materials. By embedding PCL fibers into Sylgard 184 and RTV 4420 during deposition, a bilayer structure was achieved without introducing additional bonding agents or interfacial layers. The bilayer structure failed within the fibrous layer rather than at the fiber–silicone interface, suggesting that fiber–silicone adhesion was stronger than the cohesive interactions between layers of the electrospun membrane. The collector material, i.e., the silicone type or aluminum, significantly affected fiber diameter and qualitatively altered bead morphology, highlighting the potential to tailor membrane structure through substrate selection and processing conditions. The formation of voltage-induced surface features in uncured silicone further illustrates how electrospinning parameters can be exploited to generate patterned fiber architectures. However, direct electrospinning onto uncured elastomers also presents challenges related to the limited electrical conductivity of many conventional elastomeric substrates and the time-dependent curing of the elastomer, which may influence fiber deposition and morphology. These substrate-dependent effects may nevertheless provide opportunities for further tailoring for fiber architecture and properties through the selection of elastomer and processing conditions. Together, these findings establish direct electrospinning as a simple, scalable, and material-efficient method for fabricating mechanically stable, cell-supporting interfaces. This approach holds considerable promise for simplifying material integration in biomedical research platforms and implantable medical devices while potentially easing regulatory and translational pathways by minimizing material complexity.

## Data Availability

The original contributions presented in this study are included in the article/Supplementary Materials. Further inquiries can be directed to the corresponding author.

## Supplementary Materials

The following supporting information can be downloaded at https://www.mdpi.com/article/10.3390/ijms27198785/s1.
