# Supplementary material for: Direct Integration of Electrospun Polycaprolactone Fibers with Polydimethylsiloxane: Influence of Elastomer Formulation on Fiber–Elastomer Adhesion
Source: Int J Mol Sci. 2026 Sep 30;27(19):8785. doi: 10.3390/ijms27198785 (PMC13665383; doi:10.3390/ijms27198785)
Supplement: Supplementary file 1 [file ijms-27-08785-s001.zip › ijms-4548933-supplementary.pdf]

Supplementary material

QQ plots and box plots were used to determine if the fiber diameters on aluminum, RTV 4420 and Sylgard 184 were normally distributed or not. Figure S1 shows QQ plots of the fiber diameters.

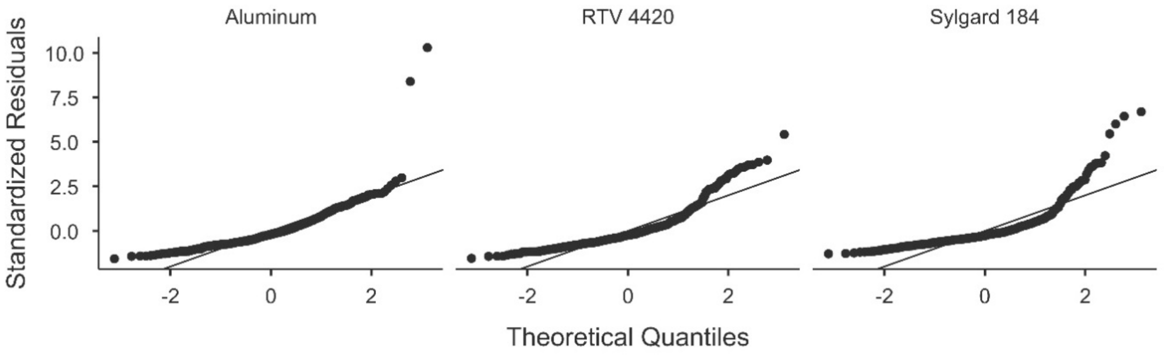

Figure S1. QQ plots of the fiber diameter of PCL on aluminum, RTV 4420 and Sylgard 184, respectively. Data is not normally distributed according to Shapiro-Wilks test ($p < 0.001$).
